# Induction of Oxidative Stress by Waterborne Copper and Arsenic in Larvae of European Seabass (*Dicentrarchus labrax* L.): A Comparison with Their Effects as Nanoparticles

**DOI:** 10.3390/toxics12020141

**Published:** 2024-02-09

**Authors:** Rafael Torronteras, Margarita Díaz-de-Alba, María Dolores Granado-Castro, Estrella Espada-Bellido, Francisco Córdoba García, Antonio Canalejo, María Dolores Galindo-Riaño

**Affiliations:** 1 Department of Integrated Sciences/Research Center RENSMA, Faculty of Experimental Sciences, University of Huelva, Avda, Tres de Marzo, s/n. Campus de El Carmen, 21007 Huelva, Spain; fcordoba@dbasp.uhu.es (F.C.G.); antonio.canalejo@dbasp.uhu.es (A.C.); 2 Department of Analytical Chemistry, Institute of Biomolecules (INBIO), Faculty of Sciences, CEI-MAR, University of Cadiz, Campus Rio San Pedro, ES-11510 Puerto Real, Spain; margarita.diaz@uca.es (M.D.-d.-A.); dolores.granado@uca.es (M.D.G.-C.); estrella.espada@uca.es (E.E.-B.); dolores.galindo@uca.es (M.D.G.-R.)

**Keywords:** arsenic nanoparticles, copper nanoparticles, environmental pollutants, fish, trace elements

## Abstract

The aim of this work was to compare the potential induction of oxidative stress and the antioxidant enzymatic response after a short-term waterborne exposure to copper (Cu) and arsenic (As) with that of the nanoparticles (NPs) of these elements (Cu-NPs and As-NPs) in fish larvae of the species *Dicentrarchus labrax*. Larvae were grouped in several tanks and exposed to different concentrations of contaminants (0 to 10 mg/L) for 24 or 96 h under laboratory conditions. Copper and arsenic concentrations were analysed in larval tissues using ICP-MS. A set of oxidative stress biomarkers, including the levels of hydroperoxides (HPs), and superoxide dismutase (SOD) and catalase (CAT) activities were assessed. The trace element concentrations (mg/kg d.w.) in larvae ranged as follows: 3.28–6.67 (Cu at 24 h) and 2.76–3.42 (Cu at 96 h); 3.03–8.31 (Cu-NPs at 24 h) and 2.50–4.86 (Cu-NPs at 96 h); 1.92–3.45 (As at 24 h) and 2.22–4.71 (As at 96 h); and 2.19–8.56 (As-NPs at 24 h) and 1.75–9.90 (As-NPs at 96 h). In Cu tests, the oxidative damage (ROOH levels) was induced from 0.1 mg/L at both exposure times, while for Cu-NPs, this damage was not observed until 1 mg/L, which was paralleled by concomitant increases in SOD activity. The CAT activity was also increased but at lower metal concentrations (0.01 mg/L and 0.1 mg/L for both chemical forms). No oxidative damage was observed for As or As-NPs after 24 h, but it was observed for As after 96 h of treatment with 0.01 mg/L. A decrease in SOD activity was observed for As after 24 h, but it turned out to be increased after 96 h. However, As-NPs did not alter SOD activity. The CAT activity was stimulated only at 96 h by As and at 24 h by As-NPs. Therefore, the two chemical forms of Cu exhibited a higher bioaccumulation and toxicity potential as compared to those of As. Importantly, the association of both Cu and As in NPs reduced the respective trace metal bioaccumulation, resulting also in a reduction in the toxic effects (mortality and biochemical). Furthermore, the assessment of oxidative stress-related biomarkers in seabass larvae appears to be a useful tool for biomonitoring environmental-occurring trace elements.

## 1. Introduction

Monitoring and evaluating pollutant contamination in aquatic systems is crucial due to its harmful effects on environmental quality and living organisms. Heavy metals and metalloids discharged into natural water end up in seawater ecosystems, affecting aquatic organisms, even at low concentrations [1]. Understanding and managing trace element contamination is essential for preserving the aquatic ecosystems [2]. Studies have shown a link between contaminated environments and fish pathologies, highlighting the sensitivity of fish to these types of pollutants [3,4].

Toxicity resulting from chronic exposure to trace elements is largely attributed to oxidative stress caused by the increase in reactive oxygen species (ROS) production [5,6]. ROS can damage cellular macromolecules, leading to significant alterations in cellular functions and cell death. Antioxidant defence mechanisms in living organisms, such as the enzymes superoxide dismutase (SOD), glutathione peroxidase (GPX), glutathione reductase (GR), and catalase (CAT), help eliminate ROS and maintain the redox balance [5]. The monitoring of oxidative damages to biomolecules and the antioxidant response in fish and shellfish provide validated early biomarkers for assessing environmental pollution and its potential impact [7,8]. Even taking into account that they are not specific for inorganic contaminants, they appear as a convenient quick starting point to identify the existence of a pollution event and to subsequently undertake complementary studies to identify and characterise the responsible toxic chemical.

Several studies have highlighted the toxicity of copper and arsenic in contaminated aquatic ecosystems, particularly affecting fish [7,9,10]. However, in environments with mixtures of pollutants, attributing specific responses to individual contaminants can be challenging. Toxicity studies in the laboratory allow for the isolation of the effects of specific elements, providing a more accurate understanding of their relationship with potentially toxic contaminants.

Copper is naturally present in aquatic ecosystems and sediments, but human activities have increased its levels to dangerous extents through mining, industrial discharges, and pesticide use [11,12]. While copper is an essential nutrient in small concentrations, exceeding certain limits makes it highly toxic to living organisms. Fish and other aquatic organisms are particularly affected by copper toxicity, which impairs salt regulation, disrupts the nervous and cardiovascular systems, and causes cell death in taste buds and olfactory sensory organs [13]. Furthermore, copper induces genotoxic effects and oxidative stress, leading to irreversible damage to DNA, proteins, and lipid membranes [14,15,16,17,18,19]. The European Commission has expanded its list of critical minerals to include copper, which is now considered as a critical and strategic metal [Annexes I and II of the Proposal for a Regulation of The European Parliament and of the Council establishing a framework for ensuring a secure and sustainable supply of critical raw materials and amending Regulations (EU) 168/2013, (EU) 2018/858, 2018/1724, and (EU) 2019/1020], which implies that it is economically and strategically important for the European economy but has a high risk associated with its supply.

Arsenic, a toxic metalloid mainly found as arsenate and arsenite in water, poses a significant risk to organisms, particularly through contaminated groundwater from mining and pesticide use [20,21]. Fish acquire arsenic through water and the food chain, leading to its accumulation in various organs [22]. Arsenic toxicity affects organisms by interacting with proteins and substituting phosphorus in biochemical reactions, damaging the bone marrow, reducing blood cells, and inhibiting enzymes such as acetylcholinesterase [23,24]. Arsenic also produces reactive oxygen species and binds to thiol groups in proteins and compounds like glutathione, leading to oxidative damage [25]. Chronic exposure to low doses of arsenic may induce lipid peroxidation, and it alters antioxidant enzyme activity and glutathione reductase activity, indicating oxidative stress during early stages of exposure [26].

According to previous studies in the literature analysing trace element concentrations in different ecosystems, the ranges of As and Cu in waters varied according to the quality of the ecosystems, establishing the following examples of intervals: from 0.5–0.8 μg/L in Algeciras Bay (Spain) [27] to <LOD to 43 mg/L in Kuwait Bay [28] for As and from 0.18–0.66 mg/L in Pasajes Harbour (Spain) [29] to 36.97–230.94 mg/L in Kakinada Bay (India) [30] for Cu. On the other hand, the Gulf of Cádiz in Spain has been historically impacted by acid mine drainage and the discharge of rivers such as the Guadiana and Guadalquivir. Metal and metalloid concentrations, including Cu, Cd, Zn, Hg, and As, are mainly associated with the Tinto and Odiel rivers, which have been affected by mining activities and flow into the Ría de Huelva. The Huelva estuary receives a daily load of 45 metric tons of heavy metals transported by the Odiel River, with the quantity and types of discharges influenced by freshwater flow and rainfall [31].

The behaviour, distribution, toxicity, and bioavailability of trace elements are highly dependent on the chemical or physical forms in which they occur. In this regard, Cu(II) and As(III) are borderline metals being intermediate elements between class A (oxygen-seeking) and class B (nitrogen/sulphur-seeking), which implies that they are able to form stable complexes with all categories of ligands [32]. Moreover, a new issue is arising with nanomaterials and nanoparticles.

The European Union defines nanomaterials as materials containing particles or constituents of nanoscale dimensions, either naturally occurring, accidental, or manufactured; these particles can be found in an unbound state or as an aggregate or agglomerate, and 50% or more of them have one or more external dimensions in the size range 1–100 nm (EUR-Reports JRC95675, 2015). Nanoparticles (NPs) are defined as having three dimensions ranging from 1 to 100 nm, derived from industrial activities, although sometimes they can offer a hydrodynamic diameter slightly greater than 100 nm [33].

The potential for pollution from nanoparticles is high due to their widespread use and increasing production [34,35]. The potential effects of nanoparticles in aquatic ecosystems depend largely on the properties of these nanoscale materials, such as their physicochemical, mechanical, and bioactive properties. Their small size and surface functionalities can increase their bioavailability, allowing their accumulation in different organs and cellular uptake [36]. Once in the cellular environment, NPs can be degraded, and their dissolution can release potentially toxic metal ions due to acidic conditions in lysosomes. The effects could be similar to those of metal ion uptake, although they may be slower in time. However, NPs can also undergo aggregation phenomena within cells that alter their molecular interactions and reduce their potential toxicity [37]. The increased bioavailability of dissolved trace elements is well known, but a full understanding of the toxicity profile of NPs is still lacking, and it is of great interest to compare the effects when trace elements occur as NPs versus metal. However, the number of studies in which both chemical forms are evaluated simultaneously is small. Furthermore, to our knowledge, there is a lack of information regarding these effects on fish in the case of Cu and As in a highly susceptible developmental stage as that of larvae.

Nanoecotoxicological studies on aquatic organisms have focused on a limited number of species such as microalgae, Daphnia, mussels, amphibians, snails, and zebrafish [35,38,39]. Studying nanomaterials is crucial to grasp their potential ecological risks. This involves assessing their behaviour, accumulation, and physical–chemical control [40]. For this, biomarkers aid in evaluating cytotoxic damage in model organisms [41]. However, there is still a limited number of studies on NPs in natural environments, particularly in aquatic ecosystems [42,43]. In particular, the generation of ROS by trace element NPs highlights the importance of evaluating ROS levels, cytotoxic effects, and antioxidant defence mechanisms in NP biomonitoring [40,44]. The information resulting from these biomarker-based studies will be of great usefulness for the agencies for environmental protection in charge of regulating and monitoring the potential ecological risk related to the presence of trace metals in aquatic systems.

Fish are good indicators of pollution due to their position in the food chain and their capacity to accumulate and respond to contaminants. The euryhaline and eurythermic seabass (*Dicentrarchus labrax*) is widespread in the Mediterranean Sea, Black Sea, and Eastern Atlantic Ocean. This species is used for human consumption as a highly prized marine fish and, as such, has significant commercial value and is widely farmed worldwide. Moreover, it proved to be valuable in monitoring studies and ecotoxicological tests to assess environmental impacts [45,46,47,48].

Therefore, the aim of this work was to compare the potential induction of oxidative stress and the antioxidant enzymatic response after a short-term waterborne exposure to copper (Cu) and arsenic (As) with that of the NPs of these elements (Cu-NPs and As-NPs) in fish larvae of the species *D. labrax*.

## 2. Materials and Methods

### 2.1. Larvae and Experimental Design

About 4000 specimens of seabass *Dicentrarchus labrax* (Linnaeus, 1758) larvae, between 0.8 and 1.0 cm in average total length and 0.075 g in average weight, were obtained from the breeding tanks of the Marine Culture Laboratory of the Andalusian Center for Marine Studies (CASEM) of the University of Cádiz, which has a seawater well. Before the experiments, larvae were acclimated under laboratory conditions with oxygen-saturated water at 20 °C and a 12/12 dark/light cycle for one week. The larvae were disease-free and did not have any history of previous chemical exposure. Larvae were fed with commercial larval feed (NIPAII). No larvae died during the acclimation process.

The bioassays were carried out by exposure to copper (Cu) and arsenic (As) and to NPs of these elements (Cu-NPs and As-NPs). The elements or species used in each bioassay were as follows: (a) the Cu exposure experiments were carried out using ionic copper; copper in complex forms is less bioavailable and less toxic than the free ionic form Cu^2+^ [49]; (b) the assays of As were carried out using arsenic trioxide because the element itself does not dissolve well in water [50]; furthermore, it is known that inorganic arsenic compounds can accumulate more in tissues than organic arsenic compounds; (c) copper-based nanoparticles were prepared as a suspension of copper oxide nanopowder (CuO) because this type of nanospecies is widely used in industrial applications and as antimicrobial and antifungal agents [51]; and (d) realgar nanoparticles prepared from As_2_S_2_ nanosuspension, which stands out for its properties as an anti-cancer agent but offers high toxicity from realgar oxidation and the release of As(III) [52,53,54]. The NP dispersions were ultrasonically prepared for 0.5 and 4 h. The following chemical reagents used to prepare the solutions for bioassays were supplied by Sigma-Aldrich: CuSO_4_·5H_2_O (99.999%; CAS No.: 7758-99-8) for Cu test; CuO nanopowder (<50 nm (TEM); CAS No.: 1317-38-0) for Cu-NP test; As_2_O_3_ (ReagentPlus^®^, ≥99.0%) for As test; and As_2_S_2_ (realgar 95%; <0.2 microns; CAS No.: 12044-30-3).

The specimens were exposed to different concentrations of each contaminant for 24 and 96 h in static tests: 0 (control), 0.01, 0.1, 1, and 10 mg/L. The concentrations were selected based on the previous literature data on the potential genotoxic effect as well as reported trace element toxicity bioassays and were within the possible values for them in real ecosystems. The bioassays were performed in duplicate, and to facilitate the task of extracting the larvae from the tanks and to avoid stress, the tests at 24 and 96 h were carried out in separate tanks, with a total number of 20 tanks per test. For each concentration and chemical species, 1.5 L plastic tanks (containers) were used, and approximately 50 *Dicentrarchus labrax* larvae were introduced into each tank (i.e., 100 larvae per treatment). The tests were carried out at the room temperature of the marine culture plant, with a 12 h photoperiod, with partial water renewal (change of 0.25 L/day with the same metal concentration and seawater matrix) and with aeration. The larvae were fed during the tests, and the amount of food supplied was weighed and controlled. Every day, the number of living and dead individuals was recorded; the latter were eliminated to avoid bacterial or fungal infection of the other fish during the breakdown. All experimental procedures were performed following the guidelines of the Directive 2010/63/EU on the protection of animals used for scientific purposes. Physical–chemical parameters of water from tanks (pH, temperature, dissolved oxygen, salinity, and total dissolved solids) were controlled daily.

After the experimental periods, larvae were harvested from each tank, washed with saline solution (9 mg/L NaCl in Milli-Q water), introduced into liquid nitrogen, and stored at −80 °C until assays. In order to have enough amount of tissue for each type of analysis, 10 larvae were taken for the biochemical analysis (in two pools of 5 units) and 40 for the trace element analysis (in two pools of about 20 individuals) from each tank. In all cases, the analyses were performed in duplicate. 

### 2.2. Physical–Chemical Parameters of Water

An electrochemical portable device (HI 9828, Hanna Instruments, Eibar, Spain) was used for the in situ measurement of different parameters: temperature, pH, dissolved oxygen (DO), salinity, and suspended solids (SSs) in the water samples. Water samples of 25 mL were taken from each tank to evaluate the dissolved organic carbon (DOC) using a TOC analyser (Analytik Jena 3100, Jena, Germany).

It should be noted that all the parameters (Table 1) are within the margins considered adequate for the survival of the species used and that they do not vary significantly throughout this study. It is noteworthy that the experiments were accomplished at thermal optimum conditions for *Dicentrarchus labrax* ranging around 20 °C, as previously defined by different authors [55].

In copper tests, the temperature varied between 16.1 and 18.9 °C; the pH was maintained between 8.1 and 9.7; dissolved oxygen varied between 6.1 and 9.6 mg/L; salinity, typical of seawater, was between 34.4 and 40.5 g/L; suspended solids were between 26.1 and 30.1 mg/L; and DOC ranged between 3.6 mg/L at 0 h and 14.7 mg/L at 96 h. Regarding the arsenic tests, the temperature values ranged between 17.0 and 20.0 °C; the pH ranged between 7.2 and 9.6; dissolved oxygen was between 6.9 and 9.4 mg/L; salinity was between 36.5 and 39.6 g/L; suspended solids were between 27.4 and 29.5 mg/L; and DOC varied between 3.6 mg/L at 0 h and 13.4 mg/L at 96 h. During all the experiments, the values of DOC slightly increased with time due to the excretions produced by the larvae and slight remains of larval feeding.

### 2.3. Trace Element Concentrations in Water

In the water samples, the amounts of trace elements were analysed to check the actual concentrations (i.e., measured concentrations). The contents of these elements were determined by two techniques depending on their concentrations: inductively coupled plasma atomic emission spectroscopy (ICP-AES) using an Iris Intrepid spectrometer with argon gas humidifier, cyclonic spray chamber (Glass), and nebuliser type SeaSprays™ (Thermo Elemental, Franklin, MA, USA) for tanks with 0.1, 1, and 10 mg/L for copper tests and 1 and 10 mg/L for arsenic tests and a Metrohm 757 VA Computrace Stand controlled by PC software 2.0 (VA Computrace, Metrohm, Zofingen, Switzerland) for lower concentrations (0 and 0.01 mg/L for copper tests and 0, 0.01, and 0.1 for arsenic tests) using differential pulse anodic stripping voltammetry (DPASV). In the case of DPASV, the samples were previously digested for 2 h using a UV digester (705 UV, Metrohm, Zofingen, Switzerland) and 50 μL of 30% H_2_O_2_ (Suprapur grade) per 15 mL of water sample. Both analytical methods were validated with certified reference materials (LGC 6016 (estuarine water), TMDA 64.2 (lake water), and BCR 610 (groundwater)). The water reference samples were analysed in triplicate, obtaining good recoveries (≥90%); blanks and limits of detection (LOD) were also evaluated (Table 2). The measured concentrations obtained have been discussed in Section 3.1, but the nominal concentrations have been used to facilitate the discussion.

### 2.4. Chemical Analysis in Larvae

Freeze-dried larvae samples (0.1–0.3 g) were acid-digested by microwave heating using 4 mL of 65% HNO_3_ (Suprapur grade) and 2 mL of 30% H_2_O_2_ (Suprapur grade). After digestion, the samples were diluted up to 25 mL with Milli-Q deionised water and analysed using ICP-MS (X7 Series plasma scan sequential inductively coupled mass spectrometer, Thermo Elemental, Winsford, UK). Analytical parameters were evaluated, and the accuracy of the applied methodology was satisfactorily evaluated using a certified reference biological material of the National Research Council Canada (NRCC): DOLT-3 (dogfish liver) (Table 3).

### 2.5. Analysis of Biomarkers

#### 2.5.1. Tissue Preparation

At the time of assay, larvae subsamples were thawed and homogenised on ice in 1.5 mL of cold 0.1 mM phosphate buffer (pH 7) containing 0.5 mM EDTA using a tissue homogeniser (IKA homogenizer 10T Basic, Staufen, Germany). Subsamples were centrifuged (Ortoarlesa Biocen 22R centrifuge, Madrid, Spain) for 60 min at 12,000× *g* (4 °C), and the supernatants were used for biochemical analyses.

#### 2.5.2. Protein Measurement

Protein quantifications were carried out according to the Bradford assay [56] using bovine serum albumin as a standard.

#### 2.5.3. Lipid Peroxidation (LPO) Assay

Lipid peroxidation was assessed by measuring the levels of lipid hydroperoxides (which result from the oxidative injury of saturated and unsaturated lipids). These were estimated according to the xylenol orange assay [57] using 0.1 mL of diluted supernatant sample (1:10 in water) and 0.9 mL of reaction mixture containing final concentrations of 25 mM H_2_SO_4_, 100 μM xylenol orange (XO), 100 mM sorbitol, and 250 μM ferrous ammonium sulphate. The pH of the reaction mixture was adjusted to 1.8 with the addition of Na_2_HPO_4_. Afterwards, the sample was centrifuged at 1000× *g* for 2 min and placed in the dark for 45 min. Absorbance was read at 560 nm. The molar extinction coefficient of 2.67 × 10^5^ M^−1^ cm^−1^ was used.

#### 2.5.4. CAT Activity Assay

Catalase (CAT) activity was determined according to the method of Claiborne [58] by measuring the decrease in H_2_O_2_ concentration in the presence of the supernatant sample at 240 nm for 2 min in phosphate buffer. Briefly, the assay mixture consisted of 1.95 mL of phosphate buffer (50 mM, pH 7), 1 mL of H_2_O_2_ (15 mM), and 0.05 mL of tissue homogenate. CAT activity was calculated in terms of nmol H_2_O_2_ consumed per min mg protein, using a molar extinction coefficient of 43.5 M^−1^ cm^−1^.

#### 2.5.5. SOD Activity Assay

Superoxide dismutase (SOD) activity was determined based on the ability of the enzyme to inhibit the reduction of nitro blue tetrazolium (NBT) [59], which was generated by xanthine oxidase. Briefly, the assay mixture consisted of 0.65 mL of phosphate buffer (50 mM, pH 7.8) with 5 mM EDTA, 0.1 mL of xanthine (50 mM), 0.1 mL of NBT (1 mM), 0.05 mL of xanthine oxidase (5.4 U mL^−1^), and a 0.05 mL aliquot of supernatant sample. The reaction was initiated by adding xanthine oxidase. The reduction of NBT by superoxide anion to blue formazan was measured at 560 nm. The rate of NBT reduction in the absence of tissue was used as the reference rate. One unit of SOD is defined as the amount of enzyme required to decrease the reference rate to 50% of maximum inhibition. The SOD activity was expressed in units per mg protein.

### 2.6. Statistical Analysis

The experiment was performed with 10 larvae for biochemical analysis (in two pools of 5) and 40 larvae for trace element analysis (in two pools of 20) per treatment tank and in duplicate (*n* = 2 for each exposure concentration), collecting four larvae pools per exposure time. Measurements from larval tissue were performed in triplicate from pools of 10 or 40 larvae. The data are expressed as mean ± SD (standard deviation). After checking normality and homoscedasticity with the Shapiro–Wilk test and the Bartlett’s test, respectively, significant differences were determined using a one-way ANOVA followed by the Duncan’s post hoc test. Significant differences were considered at *p* < 0.05. Statistical analyses were performed using the Statistical Package for Social Sciences, IBM SPSS v. 29.0 for Windows (SPSS Inc., Chicago, IL, USA).

## 3. Results

Both copper (Cu) and Cu-NP tests resulted in total lethality in the tanks with larvae exposed to the maximum concentration (10 mg/L) at both exposure times (24 and 96 h). On the other hand, treatment with 1 mg/L for the Cu test, but not for Cu-NP test, caused total lethality after 96 h of exposure. No mortality was observed in the remaining tanks with lower concentrations of copper. Regarding arsenic tests (As and As-NP tests), no lethal effect was found for all exposure concentrations.

### 3.1. Trace Element Concentrations in Water

In order to check the evolution of the measured trace element concentrations in the water of the tanks throughout the experimental assays, water samples were analysed with ICP-AES or DPASV at 0, 24, and 96 h. The data on the concentrations in water from each exposure test are shown in Table 4. Also, the deviation from the nominal concentrations over time in percentage has been included in the table.

The measured concentrations of trace elements in the seawater medium in acute toxicity tests usually deviate from the nominal concentration for several reasons, such as the following:

– The acid–base behaviour of the trace elements in seawater.
– The salinity and the high ionic strength in seawater also provide rapid aggregation [60].
– The adsorption of the trace elements on the suspended matter with subsequent precipitation.
– The dynamic behaviour of nanoparticles (due to their surface properties) such as sedimentation, flotation, or aggregation and which effects depend on the particle’s size.
– The presence of organic colloids in the aqueous environment can overcome the aggregation process of NPs in real water, such as seawater, due to the presence of counter ions in the aquatic environment, especially for NPs of metal oxides [61].
– The interactions of food with exposure to trace elements in the same way as other suspended matter. However, the solid particles may also be uptaken by the organisms.

The controversy between the use of nominal and measured concentrations is mentioned in the literature, and it is advisable to indicate both [62].

The values of the deviations of the measured concentrations from the nominal concentrations were in the following ranges: (a) Cu test: 9.5–59.6% (corresponding to the per-centage of 3.5–12.1% to SD of measurements); (b) Cu-NP test: 5.5–80.2% (corresponding to the percentage of 5.0–6.7% to SD of measurements); (c) As test: 10.5–53.2% (corresponding to the percentage of 2.9–20.3% to SD of measurements); and (d) As-NP test: 16.2–43.8% (corresponding to the percentage of 4.2–11.3% to SD of measurements). These deviations among the measured concentrations from the nominal concentrations were sometimes above the 20% recommended for toxicity tests [63]. These relative values of deviation should be considered, but the comparisons among the measured concentration at 0 h with 24 h and 96 h in absolute value are also useful to check the evolution of the trace elements in the water.

These differences among the nominal concentrations and the measured concentrations have been explained in the literature. The precipitation of amorphous Cu hydroxy compounds of chloride, carbonate, and sulphate has been described as the reason for the decrease in the measured concentrations in the Cu toxicity test in seawater [64]. The effect of microscale aggregates in the Cu-NP test contributed to this decrease [60]. The decrease in the measured concentrations was less for the arsenic tests, principally at concentrations of 0.01 and 0.1 mg/L. The adsorption of As on the suspended solids by the effect of the high ionic strength has been described [65]. In the As-NP experiments, the concentration of the metalloid measured in water also decreased to a lesser extent than in the Cu-NP tests. The arsenic nanoparticles may aggregate in the marine matrix over time like other nanoparticles, but arsenic sulphide NPs, especially amorphous ones such as realgar, may also destabilise and dissolve at the pH of seawater [66], being in the column of water.

### 3.2. Chemical Analysis in Larval Tissues

**Figure 1:**
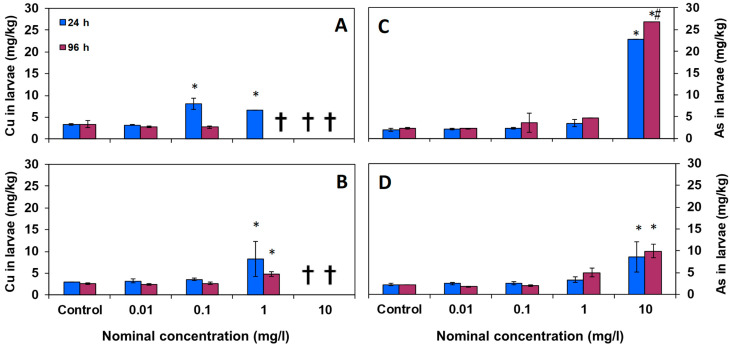
Mean values of copper and arsenic concentrations (mg/kg) in larvae of *D. labrax* with respect to the nominal concentration in water, after 24 and 96 h of exposure. (**A**) Cu, (**B**) Cu-NPs, (**C**) As, and (**D**) As-NPs (data are shown as means ± SD; symbols indicate significant differences as follows: (*) *p* < 0.05 vs. control; (#) *p* < 0.05 vs. 24 h). The crosses indicate that all larvae died at this concentration.

The levels of copper and arsenic accumulation were also measured in larvae. The mean concentrations accumulated in the larvae of *D. labrax* after 24 and 96 h exposure for each test are shown in Figure 1. In general, it can be observed that by increasing the concentration of pollutants in the water, their concentration in larvae also increased notably.

Figure 1A shows that copper in the Cu tests appeared to be significantly accumulated in larval tissues from 0.1 mg/L at 24 h, while in the Cu-NP tests (Figure 1B), the accumulation was obtained from 1 mg/L at 24 h.

On the other hand, arsenic significantly accumulated in larvae at 10 mg/L with a very high concentration after the 96 h treatments, both in the As and As-NP tests (Figure 1C,D). The exposure to As-NPs resulted in a lower accumulation in larval tissue than As.

Regarding the time of exposure of the larvae to the pollutants, it was observed that the concentration of copper found in larvae did not increase significantly with time. Conversely, in the case of arsenic, the concentrations found in larvae were slightly higher at 96 h than at 24 h.

### 3.3. Lipid Peroxidation

**Figure 2:**
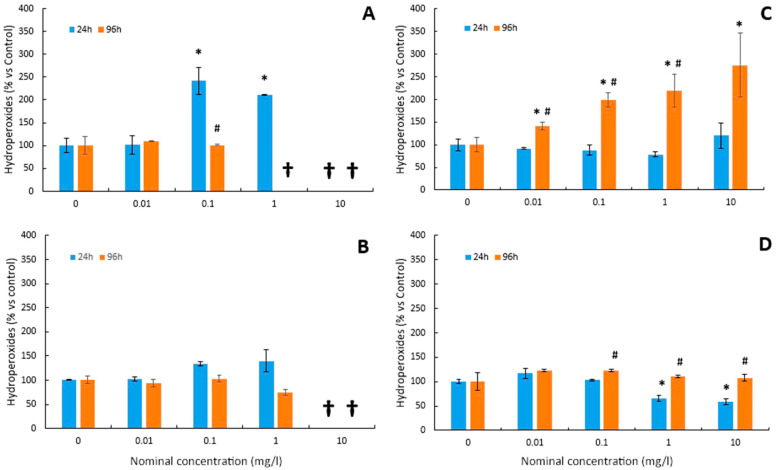
Mean values of hydroperoxides in larvae of *D. labrax* after 24 and 96 h exposure to (**A**) Cu, (**B**) Cu-NPs, (**C**) As, and (**D**) As-NPs (data are shown as means ± SD versus controls; symbols indicate significant differences as follows: (*) *p* < 0.05 vs. control; (#) *p* < 0.05 vs. 24 h). The crosses indicate that all larvae died at this concentration.

The levels of lipid peroxidation (LPO) in the larvae of *D. labrax* after the bioassays are shown in Figure 2A,B for Cu and Cu-NPs, respectively, and in Figure 2C,D for As and As-NPs, respectively. Both Cu (Figure 2A) and Cu-NPs (Figure 2B) increased hydroperoxides from 0.1 mg/L at 24h. However, no effects were observed at 96 h for both chemical forms. In the case of the As test, no oxidative damage was detected after 24 h exposure (Figure 2C). However, LPO was induced from the lowest concentration (0.01 mg/L) after 96 h (Figure 2C). No effects were observed for As-NPs (Figure 2D) with respect to the control group after 96 h.

### 3.4. Superoxide Dismutase Activity

**Figure 3:**
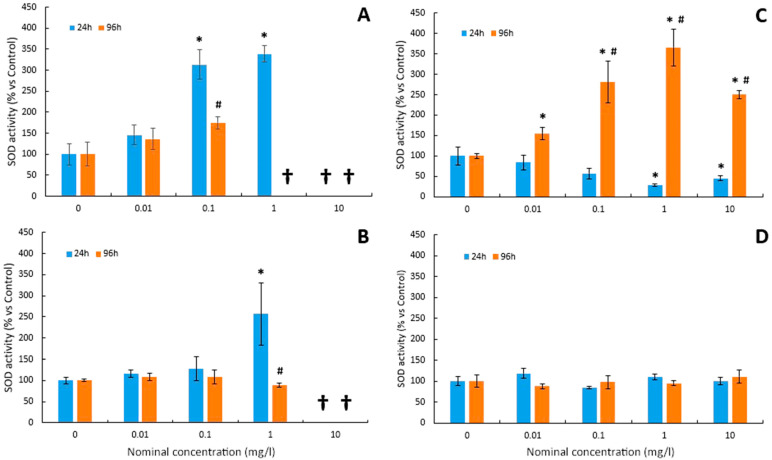
Mean values of SOD activity in larvae of *D. labrax* after 24 and 96 h exposure to (**A**) Cu, (**B**) Cu-NPs, (**C**) As, and (**D**) As-NPs (data are shown as means ± SD versus controls; symbols indicate significant differences as follows: (*) *p* < 0.05 vs. control; (#) *p* < 0.05 vs. 24 h). The crosses indicate that all larvae died at this concentration.

The variations in the SOD activity before and after exposure to Cu and Cu-NPs are depicted in Figure 3A,B, respectively. For the Cu test, superoxide dismutase (SOD) activity paralleled concomitant increases in hydroperoxide levels. After 24 h of exposure to Cu, a significant increase in the SOD activity was observed at concentrations of 0.1 and 1 mg/L (Figure 3A). Similar results, although not significant, were obtained after 96 h of treatment (Figure 3A).

Regarding Cu-NPs, SOD activity was only significantly stimulated with the maximum sublethal dose (1 mg/L) after 24 h of exposure (Figure 3B). However, no significant changes were observed with respect to the control group in any of the treated groups after 96 h (Figure 3B).

Superoxide dismutase (SOD) activity decreased in As tests after 24 h but increased after 96 h (Figure 3C). As shown in Figure 3C, SOD activity showed a tendency towards a dose-dependent decrease after 24 h of exposure to the metalloid. Thus, exposure to higher As concentrations caused an inhibition of SOD activity in larvae (Figure 3C). The reduction in SOD activity levels became greater as the arsenic doses increased, which were only significant at the highest concentrations (1 and 10 mg/L). Conversely, SOD activity increased in parallel with the increase in As dose, although the maximum level of activity was reached with an As concentration of 1 mg/L (Figure 3C).

In the case of the As-NP test (Figure 3D), the different concentrations for 24 h and 96 h did not significantly alter the SOD activity compared to the control group.

### 3.5. Catalase Activity

**Figure 4:**
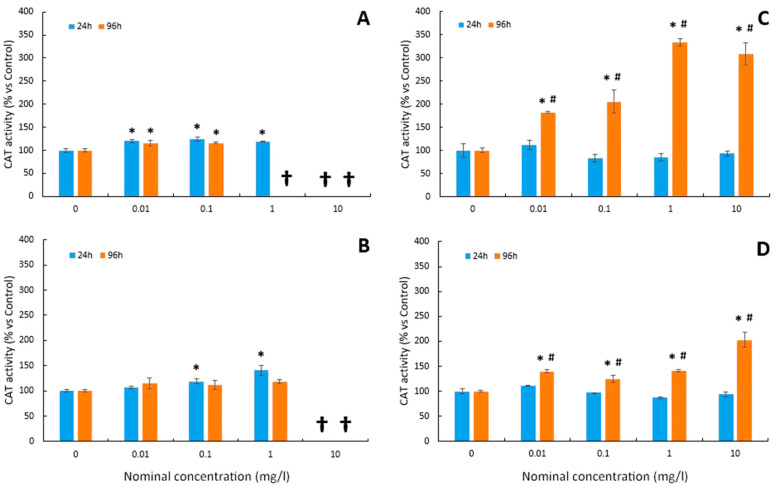
Mean values of CAT activity in larvae of *D. labrax* after 24 and 96 h exposure to (**A**) Cu, (**B**) Cu-NPs, (**C**) As, and (**D**) As-NPs (data are shown as means ± SD versus controls; symbols indicates significant differences as follows: (*) *p* < 0.05 vs. control; (#) *p* < 0.05 vs. 24 h). The crosses indicate that all larvae died at this concentration.

For Cu tests, as shown in Figure 4A, the different exposure concentrations were able to significantly increase the CAT activity in larvae. This activity was significantly increased from the lowest dose (0.01 mg/L) both at short- (24 h) and long-term (96 h) exposure. The CAT enzyme, therefore, seems to be more sensitive to the presence of this pollutant (Figure 4A) than the superoxide dismutase enzyme (Figure 3A). In the case of Cu-NP tests (Figure 4B), CAT activity was also significantly increased at 0.1 mg/L and 1 mg/L at 24 h. However, no effects were observed at 96 h of exposure (Figure 4B).

In the case of arsenic tests, the CAT activity did not show significant differences compared to the control in both As and As-NPs at 24 h exposure (Figure 4C,D). Conversely, the CAT activity was significantly stimulated by both As and As-NPs at 96 h (Figure 4C and Figure 4D, respectively), with a significant induction of this enzymatic activity from the lowest concentration (0.01 mg/L).

## 4. Discussion

In the present work, parallel studies were carried out on the toxic effect and the induction of oxidative stress caused by the presence of copper (Cu) and arsenic (As) and the NPs of these elements (Cu-NPs and As-NPs) in seabass larvae, allowing for a good comparison between two different pollutants and two chemical forms. In order to avoid possible alterations that could cause stress in individuals, a static model was used, evaluating a wide range of environmentally relevant concentrations in accordance with previous studies assessing Cu and As concentrations in different aquatic ecosystems [27,28,29,30]. As a whole, the two chemical forms of Cu exhibited a higher bioaccumulation and toxicity as compared to those of As. Importantly, the association of both Cu and As in NPs reduced the respective trace metal bioaccumulation, resulting also in a reduction in the toxic effects (mortality and biochemical).

In the Cu tests, the exposure of the larvae to the pollutant caused total lethality in the groups treated at doses of 10 mg/L at 24 h and 96 h and in the 1 mg/L group only in the 96 h tests. These results demonstrated the high short-term toxic potential of copper on the larval stages of *D. labrax*, which has previously been documented for other fish species. Leite et al. [17] also found 75–80% mortality in zebrafish (*Danio rerio*) larvae exposed to 1.6 mg/L copper after 24 h of exposure and 15–20% mortality with a copper dose of 0.64 mg/L, while no lethality was observed at lower concentrations. On the other hand, long-term exposure to the metal induced lethality at lower concentrations. According to Hansen et al. [67], the presence of copper at a concentration of 0.05 mg/L for 56 days caused the death of 47.8% of juvenile rainbow trout (*Oncorhynchus mykiss*).

Lipid peroxidation (LPO) is considered a good indicator of cell damage caused by oxidative stress [68]. In this study, the levels of hydroperoxides (ROOH) have been quantified to assess oxidative damage in cell membranes. ROOHs are early products of the oxidative attack on lipids that occurs during lipid peroxidation of membranes. After exposing the larvae to Cu for 24 h, the levels of hydroperoxides increased significantly. However, for 96 h tests, no significant changes were found. The results of this study at 24 h are in agreement with previous studies where the increase in copper in the medium resulted in a stimulation of LPO in different organs [14,15,16].

SOD is considered the first line of defence against oxidative stress, catalysing the dismutation of the superoxide radical to H_2_O_2_ and O_2_. In this study, SOD activity was stimulated at low concentrations of Cu in the medium, as previously observed in *Gasterosteus aculeatus* [69] and zebrafish [18]. In fact, an increased SOD activity has been proposed as a tolerance mechanism to counteract the oxidative stress induced by trace elements in fish [70,71]. However, a decrease in SOD activity caused by an increase in copper in the medium has also been described in zebrafish larvae [17].

CAT activity also plays a key role in ROS detoxification. In this study, the CAT activity was also stimulated after copper exposure at both 24 h and 96 h. Likewise, the stimulation occurred from the lowest concentrations (0.01 mg/L), indicating that CAT activity was more sensitive to the presence of Cu than SOD activity. Similarly, CAT activity was induced in the liver of *Poecilia viviparous* and in stickleback after 96 h of exposure to low copper concentrations [16]. On the other hand, the inhibition of CAT activity by increasing copper in the medium has also been described in the adults [18] and larvae of zebrafish [17]. Indeed, in *Oreochromis niloticus*, Atli et al. [19] found that the activating or inhibitory effect of Cu on CAT activity after 96 h was dependent on the organ analysed.

There are key chemical and behavioural differences between the exposure to Cu or As and that of the Cu-NPs and As-NPs, highlighting the following: (a) bioavailability, (b) solubility, (c) the way in which they are absorbed and excreted, and (d) even the lethal and sublethal effects on aquatic biota. It is known that the seawater matrix and the ionic strength affect the stability, solubility, dissociation, and agglomeration of NPs [34]. Nevertheless, an important factor concerning the potential for nanoparticles based on trace elements to induce adverse effects is related to their bioavailability [34], and the bioavailability and solubility of NPs in water are lower. In fact, it has been suggested that copper nanoparticles (Cu-NPs) dissolve and release the metallic ions that they carry in their structure more slowly, taking hours or days to do so [72]. However, the behaviour of the NPs can differ depending on the trace element on which they are based. Indeed, in our study, Cu-NPs and As-NPs showed different aqueous behaviour, as described above in Section 3.1. This aqueous chemical behaviour of NPs determines their effects after exposure. Regarding the induction of lethality by nanoparticles, the literature indicates that it occurs at concentrations of the order of mg/L, whereas many of the dissolved metals, such as copper, cause lethality at concentrations of µg/L, suggesting that the ionic form may be more toxic than the nanoparticle form [73]. On the other hand, the absorption and entry of trace elements into aquatic organisms should also be taken into account. Ions enter the body of aquatic animals through transmembrane ion transport proteins, while the absorption of NPs seems to be carried out through endocytosis mechanisms. In the theoretical model proposed for the absorption of NPs in fish, it is shown that, due to the hydrophilic nature of the NPs, the mucous secretion emitted by the gills is capable of trapping them and facilitating their passage through the epithelial lipid membrane. Its penetration into the cell interior involves vesicular transport through the endoplasmic reticulum (ER) and the Golgi apparatus [74]. In fish, NPs can interact primarily through the skin, gills, and/or intestine via the diet as particles. Alternatively, NP uptake may occur indirectly through the release of free metal ions within the superficial microlayer of the cell membrane where the trace element NP was previously associated with the cell surface [75]. It has also been suggested that the excretion processes of ions and nanoparticles are different. Fish normally excrete metal ions via the liver, with small urinary losses [76], and via the gill ion flow, whereas the excretion of NPs in fish is likely to be via the kidneys. All this may lead to different behaviour and effects between the exposure to trace elements (Cu or As) and their (Cu- and As-) based nanoparticles.

In this study, seabass larvae were highly sensitive to the presence of Cu-NPs since the highest concentration tested (10 mg/L) caused lethality at both 24 and 96 h. However, Cu tests turned out to be more toxic since mortality was detected at 96 h at concentrations of an order of magnitude lower (1 mg/L). These results agree with those of previous studies. Thus, Shaw et al. [77] observed in juvenile rainbow trout that at 96 h there was a mortality of 85% for individuals exposed to 100 µg/L copper sulphate and 20% for those exposed to copper nanoparticles. On the other hand, several authors have reached the conclusion that the size of the NPs is very important with regard to their toxicity. Likewise, in zebrafish embryos exposed to copper nanoparticles of 25, 50, and 100 nm in size, the referenced LC50s were 0.58, 1.65, and 1.90 mg/L, respectively. In this same study, the LC50 for embryos exposed to copper nitrate was also found to be 0.70 mg/L. In this way, it was indicated that copper in its soluble form is more toxic than in NP form and that, in turn, the toxicity of copper nanoparticles increases as the size of the particles is reduced [73].

Existing data regarding the ecotoxicology of NPs are very limited, but even so, it has been suggested that most of the toxicity of trace element NPs lies in their ability to induce oxidative stress [40,44,78,79]. In this study, the induction of oxidative stress by Cu-NPs was also observed. Thus, a significant increase in hydroperoxide levels was detected in the 24 h tests. On the contrary, in 96 h tests, no significant changes were observed in the levels of hydroperoxides. Comparing these results with those obtained for the Cu test, which induced a significant increase in LPO levels at lower concentrations, it can be concluded that, for seabass larvae, Cu causes more oxidative damage than Cu-NPs. Similar results were found in previous studies. According to Shaw et al. [77], the soluble form of copper induced a greater increase in lipid peroxidation levels than its nanoparticulate counterpart. On the other hand, the behaviour of the analysed antioxidant enzymes (SOD and CAT) is qualitatively reminiscent of that already shown for Cu. Both showed an increase in SOD and CAT activity with a dose-dependent trend at 24 h, but no significant changes in activity levels were observed in the 96 h tests. This observed increase in CAT activity may be due to the antioxidant defence system compensating for the decrease in SOD.

As a whole, the results obtained in our work suggest that copper in any of its forms (Cu or Cu-NPs) is highly toxic for seabass larvae, with copper ions being more harmful. In the present study, the As-NPs did not induce any lethal effect at the concentrations tested. Establishing a relationship between the results obtained for the different biomarkers tested, it can be suggested that seabass larvae develop a certain tolerance to the presence of As-NPs in the water, largely avoiding oxidative damage thanks to the increased CAT activity.

In As tests, no lethality was found in any of the treatments. These results agree with some median lethal concentrations (LC50) found in other species, ranging from 42 to 84 mg/L after 96 and 48 h of exposure [23,26].

In this study, exposure to As for 24 h did not cause an increase in ROOH, but both SOD and CAT activities were inhibited. Similarly, Ventura-Lima et al. [80] found that the reduction in CAT and SOD activity was not accompanied by changes in LPO levels. On the other hand, the data on hydroperoxides found in this study in response to As exposure for 96 h coincide with those described in gills of rockfish (*Sebastes schlegelii*) exposed to increasing concentrations of arsenic in which a parallel increase in the levels of CAT, SOD, and LPO was observed [81].

In our study, at 24 h the SOD activity was decreased at the highest concentrations, while at 96 h, it was stimulated from a low concentration such as 0.1 mg/L. In previous studies, disparate results have been shown in the response pattern of SOD activity to arsenic. Thus, Altikat et al. [82] observed in *Cyprinus carpio carpio* exposed for a month to arsenic concentrations of 0.5 and 1 mg/L that the SOD response was dependent on the organ analysed. Bhattacharya and Bhattacharya [26] reported that SOD activity increased in parallel to H_2_O_2_ levels in the cell cytoplasm, after only 24 h of exposure to As. Likewise, it has been proven that chronic exposure to small concentrations of arsenic can cause an increase in the levels of SOD mRNA accompanied by an increase in its enzymatic activity [83]. The increase in the levels of H_2_O_2_ and other ROS in the peroxisomes and the cytoplasm can lead to the stimulation of SOD activity by the effect of arsenic, but, on the other hand, the binding of arsenic cations to the SH groups of SOD can cause a reduction or even inhibition in activity [26].

Regarding the CAT activity, it did not present significant variations in response to the different concentrations of As applied during 24 h. On the contrary, the activity was significantly stimulated from the lowest dose of As tested at 96 h. These changes in the enzymatic response suggest that, at certain concentrations, the regulation of CAT is more conditioned by the time of exposure to As than by its concentration. Previous studies have found different CAT responses. In adults of *Cyprinus carpio carpio* exposed to arsenic concentrations of 0.5 and 1 mg/L, the CAT activity decreased in a dose-dependent manner in the organs analysed, with the exception of muscle tissue [82]. In contrast, in adult *Clarias batrachus* exposed to sublethal concentrations of the metalloid for 10 days, the CAT activity increased in liver tissue [26], while changes were not observed in the gills of juvenile *Danio rerio* after 48 h [80].

Taken together, the results suggest that when exposure to As is short (24 h), *Dicentrarchus labrax* larvae show some tolerance to this pollutant. However, in the longer term (96 h), As induces a level of oxidative stress that cannot be counteracted even with a significant increase in the enzymatic antioxidant response. Furthermore, in our study, As seems to be less toxic than Cu, which could be related to the higher bioaccumulation levels observed for Cu than for As.

When comparing the effect observed in the As test, which induced a strong oxidative stress response, with that of As-NPs, it should be noted that the latter showed a very modest response. Interestingly, this is likely to be related to a reduced level of As accumulation in the larval bodies. In fact, lipid peroxidation was not induced by As-NPs, which did not induce any change in SOD activity either. However, the CAT activity was increased after 96 h of exposure, which could have a key role in precluding the oxidative damage in the membrane lipids. It can be established, therefore, that As is more toxic to *D. labrax* larvae than its nanoparticle form. On the other hand, as compared to the Cu-NPs, As-NPs were also demonstrated to produce lower levels of trace metal bioaccumulation, which could account for their lower toxicity.

## 5. Conclusions

Altogether, our results show a differential acute induction of oxidative damage and of an enzymatic antioxidant response for both copper and arsenic in their different chemical forms. The two forms of Cu produced a higher bioaccumulation and toxicity as compared to those of As. Significantly, the association of both Cu and As in NPs reduced the respective trace metal bioaccumulation, resulting also in a reduction in the toxic effects (mortality and biochemical). In addition, the evaluation of oxidative stress-related biomarkers in seabass larvae seems to be a useful tool for the biomonitoring of environmental-occurring trace elements.

## Figures and Tables

**Table 1:** Average values of physical–chemical parameters in water (data are expressed as means ± SD from all tanks for each test).

Test	T (°C)	pH	O_2 dissolved_	Salinity	Suspended Solids	DOC
			(mg/L)	(g/L)	(mg/L)	(mg/L)
Cu	17.4 ± 1.0	8.8 ± 0.4	8.0 ± 0.8	38.8 ± 0.7	29.0 ± 0.5	6.8 ± 3.1
Cu-NP	17.6 ± 0.8	8.8 ± 0.2	7.9 ± 0.6	38.3 ± 1.5	28.7 ± 1.0	7.9 ± 3.6
As	17.5 ± 0.6	8.3 ± 0.5	7.6 ± 0.6	38.6 ± 0.7	28.9 ± 0.4	8.0 ± 2.5
As-NP	17.6 ± 0.5	8.5 ± 0.3	7.7 ± 0.7	38.4 ± 0.8	28.7 ± 0.5	6.7 ± 2.4

**Table 2:** Analytical quality control data for copper and arsenic analysis in water (*n* = 10 for LOD; *n* = 3 for analysis of reference material; data are expressed as means ± SD).

Element	Reference Material	Method	LOD of Method (µg/L)	Blank Concentration (µg/L)	Found Concentration (µg/L)	Certified Concentration (µg/L)
Cu	LGC 6016	DPASV	0.069	1.944	189.3 ± 3.2	190.0 ± 2.0
Cu	LGC 6016	ICP-AES	6.300	<LD	189.3 ± 3.8	190.0 ± 2.0
As	BCR-610	DPASV	0.098	<LD	9.61 ± 0.92	10.8 ± 0.4
As	TMDA-64.2	ICP-AES	65.000	<LD	158.4 ± 24.0	162.0 ± 7.7

Low-density polyethylene bottles (LDPE, Nalgene) and polystyrene flasks were acid-cleaned and used to store samples and reagent solutions. All the clean material was kept in plastic bags to avoid lab air pollution. Water was purified by reverse osmosis with an Elix 3 (Milli-RO) system followed by ion exchange with an 18 MΩ cm deionised Milli-Q50 System (Millipore, Burlington, MA, USA).

**Table 3:** Analytical quality control data for copper and arsenic analysis in larvae (*n* = 10 for LOD; *n* = 3 for analysis of reference material; data are expressed as means ± SD).

Element	LOD of Method (µg/L)	Blank Concentration (µg/L)	Found Concentration (mg/kg)	Certified Concentration (mg/kg)
Cu	0.004	0.023	31.7 ± 0.6	31.2 ± 1
As	0.293	<LD	10.6 ± 0.1	10.2 ± 0.5

**Table 4:** Copper and arsenic measured concentration (mg/L) in tanks during experiments (data are expressed as means ± SD from two replicate tanks for each treatment; the crosses indicate that all larvae died at this concentration; values in parentheses are the percentage deviation of nominal concentrations from measured concentrations).

Nominal Concentration	Copper Test	Exposure Time			Arsenic Test	Exposure Time		
		0 h	24 h	96 h		0 h	24 h	96 h
Control	Cu	0.001 ± 0.001	0.001 ± 0.002	0.002 ± 0.001	As	0.002 ± 0.001	0.002 ± 0.001	0.004 ± 0.003
	Cu-NP	0.000 ± 0.000	0.000 ± 0.000	0.002 ± 0.002	As-NP	0.002 ± 0.000	0.002 ± 0.000	0.002 ± 0.000
0.01	Cu	0.008 ± 0.001 (−24.7%)	0.006 ± 0.000 (−36.2%)	0.004 ± 0.001 (−59.6%)	As	0.009 ± 0.001 (−13.5%)	0.009 ± 0.000 (−13.5%)	0.009 ± 0.001 (−14.5%)
	Cu-NP	0.005 ± 0.001 (−51.8%)	0.003 ± 0.000 (−67.5%)	0.002 ± 0.001 (−80.2%)	As-NP	0.007 ± 0.001 (−25.4%)	0.008 ± 0.001 (−25%)	0.007 ± 0.001 (−26%)
0.1	Cu	0.131 ± 0.009 (31.3%)	0.117 ± 0.003 (17%)	0.080 ± 0.000 (−20%)	As	0.058 ± 0.037 (−42.4%)	0.078 ± 0.003 (−22.4%)	0.110 ± 0.021 (10.5%)
	Cu-NP	0.106 ± 0.008 (5.5%)	0.081 ± 0.006 (−19.5%)	0.068 ± 0.004 (−31.7%)	As-NP	0.084 ± 0.008 (−16.2%)	0.077 ± 0.020 (−23.3%)	0.082 ± 0.006 (−17.9%)
1	Cu	0.905 ± 0.070 (−9.5%)	0.643 ± 0.000 (−35.7%)	**†**	As	0.865 ± 0.017 (−13.5%)	0.730 ± 0.047 (−27%)	0.595 ± 0.023 (−40.6%)
	Cu-NP	0.712 ± 0.041 (−28.9%)	0.335 ± 0.065 (−66.5%)	0.222 ± 0.045 (−77.8%)	As-NP	0.769 ± 0.020 (−23.1%)	0.563 ± 0.096 (−43.8%)	0.733 ± 0.010 (−26.8%)
10	Cu	8.513 ± 1.213 (−14.9%)	**†**	**†**	As	5.640 ± 1.006 (−43.6%)	6.415 ± 1.902 (−35.9%)	4.681 ± 0.283 (−53.2%)
	Cu-NP	7.910 ± 0.561 (−20.9%)	**†**	**†**	As-NP	7.628 ± 0.276 (−23.7%)	6.232 ± 1.844 (−37.7%)	6.911 ± 0.691 (−30.9%)

## Data Availability

Data is contained within the article.
